# Visualization of Gap Junction–Mediated Astrocyte Coupling in Acute Mouse Brain Slices

**DOI:** 10.21769/BioProtoc.5220

**Published:** 2025-02-20

**Authors:** Nine F. Kompier, Gabrielle Siemonsmeier, Niklas Meyer, Helmut Kettenmann, Fritz G. Rathjen

**Affiliations:** 1 Cellular Neurosciences, Max Delbrück Center for Molecular Medicine, Berlin, Germany; 2 Shenzhen Institutes of Advanced Technology, Chinese Academy of Sciences, Shenzhen, China; 3 Developmental Neurobiology, Max Delbrück Center for Molecular Medicine, Berlin, Germany

**Keywords:** Astrocytes, Panglial networks, Cell-to-cell coupling, Gap junctions, Patch-clamp, Biocytin, Dialysis, Sulforhodamine 101, Immunohistochemistry

## Abstract

Gap junctions are transmembrane protein channels that enable the exchange of small molecules such as ions, second messengers, and metabolites between adjacent cells. Gap junctions are found in various mammalian organs, including skin, endothelium, liver, pancreas, muscle, and central nervous system (CNS). In the CNS, they mediate coupling between neural cells including glial cells, and the resulting panglial networks are vital for brain homeostasis. Tracers of sufficiently small molecular mass can diffuse across gap junctions and are used to visualize the extent of cell-to-cell coupling in situ by delivering them to a single cell through sharp electrodes or patch-clamp micropipettes. Here, we describe a protocol for pre-labeling and identification of astrocytes in acute mouse forebrain slices using Sulforhodamine 101 (SR101). Fluorescent cells can then be targeted for whole-cell patch-clamp, which allows for further confirmation of astroglial identity by assessing their electrophysiological properties, as well as for passive dialysis with a tracer such as biocytin. Slices can then be subjected to chemical fixation and immunostaining to detect dye-coupled networks. This protocol provides a method for the identification of astrocytes in live tissue through SR101 labeling. Alternatively, transgenic reporter mice can also be used to identify astrocytes. While we illustrate the use of this protocol for the study of glial networks in the mouse brain, the general principles are applicable to other species, tissues, and cell types.

Key features

• Pre-labeling of live astrocytes in acute adult mouse brain slices using the dye Sulforhodamine 101.

• Dialysis of biocytin into individual astrocytes using whole-cell patch-clamp electrophysiology.

• Staining of biocytin by streptavidin and immunostaining of GFAP, imaging, and analysis of dye-coupled astrocytic networks.

• Can be used for other glial cell types and might be adapted to other tissues and species.

## Graphical overview


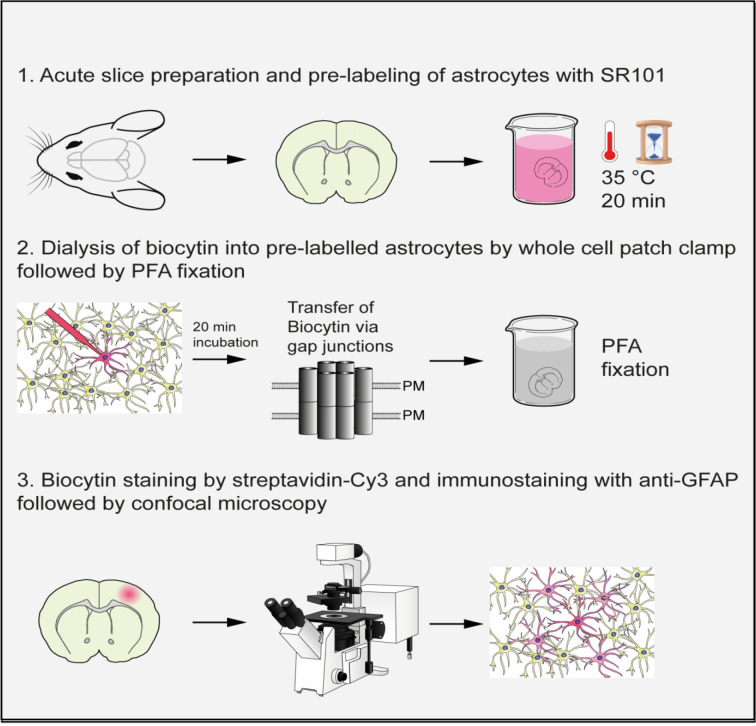


## Background

Gap junctions are transmembrane protein channels that allow for the exchange of ions and small molecules, including second messengers and metabolites, between adjacent cells [1–4]. These channels are formed by a protein family called connexins that is found in various mammalian organs, including the skin, endothelium, liver, pancreas, muscle, and central nervous system (CNS) [5–7]. In the CNS, gap junctions facilitate communication between neural cells, particularly astrocytes that form large networks. Gap junction coupling between identified astrocytes has first been described in primary mouse cultures. After injection of the gap junction–permeable dye Lucifer Yellow into one astrocyte, the dye spread into the network of adjacent cells. After subsequent labeling of the cells with the astrocyte-specific marker glial fibrillary acidic protein (GFAP), it was evident that the dye had spread within the astrocyte cell population [8]. The first evidence for coupling between identified glial cells in an acute slice preparation was obtained in the cerebellum [9]. Interastrocytic, interoligodendrocytic, and astro-oligodendrocytic coupling has been shown in situ in both grey and white matter by dye-coupling experiments through dialysis of a gap-junction permeable dye or tracer into one cell through sharp electrodes or patch-clamp pipettes [10–13]. By contrast, microglia are not incorporated into gap-junction coupled networks [14,15]. The size, shape, and composition of coupled networks are highly variable depending on the region analyzed [12,13,16,17] and are altered in pathological conditions, for example in mouse models of Alzheimer’s disease [18] and temporal lobe epilepsy [19,20].

In situ identification of cells for dye-coupling experiments often relies on transgenic reporter mice expressing fluorescent proteins under cell type–specific promoters to identify cells of interest [12,13,16,17]. However, these lines can be expensive to maintain or have to be crossbred if studies require other genetic manipulations. Moreover, some reporter lines show ectopic expression, such as astrocyte reporter lines labeling NG2 glia in some regions [17,21,22].

Sulforhodamine 101 is a non-toxic dye that is preferentially taken up by astrocytes upon topical application [23,24]. It is therefore a quick and inexpensive alternative to identify astrocytes and can be applied in any mouse model of interest. Using a patch-clamp rather than sharp electrodes to passively dialyze SR101-positive cells with a gap junction–permeable dye or tracer such as biocytin, a simple voltage step protocol can be performed to confirm astrocyte identity. In addition, the patch-clamp allows the researcher to monitor cellular health and the quality of the dialysis process. Post-hoc staining and imaging of biocytin diffusion allows for the reliable detection and characterization of dye-coupled cells. Different tracers of different molecular masses and charges, such as Lucifer Yellow (457.25 Da; charge -2 at neutral pH), ethidium bromide (394.32 Da; charge +1), and biocytin (372.47; net charge: 0) have different diffusion rates [25,26], which should be taken into account to allow for comparison between studies. Biocytin, the tracer used in this protocol, is a derivative of biotin. It has strong affinity for avidin or streptavidin and can therefore easily be visualized in brain slices. Neurobiotin, another biotin derivative, has a higher solubility but a net charge of + 1 and thus a different diffusion rate than biocytin.

## Materials and reagents

**Reagents**

1. Sucrose (Sigma-Aldrich, catalog number: 4621.2)

2. NaHCO_3_ (Sigma-Aldrich, catalog number: 6885.1)

3. KCl (Sigma-Aldrich, catalog number: 6781.1)

4. NaH_2_PO_4_·H_2_O (AppliChem, catalog number: 1319651211)

5. MgSO_4_ (Sigma-Aldrich, catalog number: 8283.2)

6. CaCl_2_ (Sigma-Aldrich, catalog number: A119.1)

7. Glucose (Sigma-Aldrich, catalog number: 6887.1)

8. NaCl (Sigma-Aldrich, catalog number: 3957.2)

9. MgCl_2_ (Sigma-Aldrich, catalog number: KK36.1)

10. K_2_HPO_4_·3H_2_O (Sigma Merck, catalog number: 1.05099.100)

11. Sulforhodamine 101 (Sigma-Aldrich, catalog number: MKBJ0694/MKCD2256)

12. EGTA (Sigma-Aldrich, catalog number: E0396-100G)

13. HEPES (Carl Roth, catalog number: 9105.3)

14. Na_2_ATP (Sigma Merck, catalog number: A7699)

15. Potassium gluconate (Sigma-Aldrich, catalog number: B4261)

16. Biocytin (Sigma-Aldrich, catalog number: 576-19-2)

17. Lucifer Yellow (Sigma-Aldrich, catalog number: L0259-100MG)

18. Paraformaldehyde (Sigma-Aldrich, catalog number: P6148-1KG)

19. Triton X-100 (Carl Roth, catalog number: 3051.3)

20. Bovine serum albumin (Carl Roth, catalog number: 0163.2)

21. Normal donkey serum (Sigma Merck, catalog number: S30-100ml)

22. Tween 20 (Carl Roth, catalog number: 9127.1)

23. DAPI (Sigma-Aldrich, catalog number: D9542)

24. Cy3-conjugated streptavidin (Dianova, catalog number: 016-160-084)

25. Guinea-pig anti-GFAP antibody (Synaptic systems, catalog number: 173004)

26. Alexa Fluor 488-conjugated donkey anti-rabbit IgG (Dianova, catalog number: 711-545-152)

27. Alexa Fluor 647-conjugated donkey anti-guinea pig IgG (Dianova, catalog number: 706-605-148)

28. Phosphate-buffered saline (PBS) (Sigma-Aldrich, catalog number: P5244)

29. Tris-buffered saline (TBS) (Sigma-Aldrich, catalog number: T5912

**Solutions**

1. Slicing solution (see Recipes)

2. Artificial cerebrospinal fluid (ACSF) (see Recipes)

3. ACSF with Sulforhodamine 101 (see Recipes)

4. Intracellular solution (ICS) with 0.5% biocytin (see Recipes)

5. Fixation solution with Paraformaldehyde (PFA) (see Recipes)

6. Blocking solution for immunohistochemistry (see Recipes)

7. Antibody incubation solution for immunohistochemistry (see Recipes)

8. Washing solution for immunohistochemistry (see Recipes)

**Recipes**

**1. Slicing solution**

*Note: Slicing solution should be maintained at 4 °C during immediate use and continuously carbonated (95% O_2_, 5% CO_2_). The desired final volume is dependent on the size of the slicing chamber and could therefore differ from 500 mL.*

**Table:** 

Reagent	Final concentration	Quantity or Volume
Sucrose	230 mM	39.3645 g
NaHCO_3_	26 mM	1.09213 g
KCl	2.5 mM	0.0932 g
NaH_2_PO_4_	1.25 mM	0.0862 g
MgSO_4_	10 mM	1.2324 g
CaCl_2_	0.5 mM	0.02775 g
Glucose dH_2_O	10 mM n/a	0.99085 g Q.S. 500 mL
Total	n/a	mL

**2. ACSF**

*Note: ACSF should be used at room temperature and continuously carbonated (95% O_2_, 5% CO_2_). It can be kept for one week at 4 °C.*

**Table:** 

Reagent	Final concentration	Quantity or Volume
NaCl	134 mM	7.83096 g
KCl	2.5 mM	0.1864 g
MgCl_2_	1.3 mM	0.1238 g
CaCl_2_	2 mM	0.22198 g
K_2_HPO_4_	1.26 mM	0.2195 g
NaHCO_3_	26 mM	2.1842 g
Glucose	10 mM	1.9817 g
dH_2_O	n/a	Q.S. 1 L
Total	n/a	1 L

**3. ACSF with Sulforhodamine 101**

*Note: ACSF with Sulforhodamine 101 should be used at 35 °C and continuously carbonated (95% O2, 5% CO2). Can be aliquoted into 250 mL aliquots, stored at -20 °C, and freshly thawed for each experiment.*

**Table:** 

Reagent	Final concentration	Quantity or Volume
NaCl	134 mM	7.83096 g
KCl	2.5 mM	0.1864 g
MgCl_2_	1.3 mM	0.1238 g
CaCl_2_	2 mM	0.22198 g
K_2_HPO_4_	1.26 mM	0.2195 g
NaHCO_3_	26 mM	2.1842 g
Glucose	10 mM	1.9817 g
dH_2_O	n/a	Q.S. 1 L
Sulforhodamine 101	1 μM	0.0006 g
Total	n/a	1 L

**4. ICS solution with 0.5% biocytin**

*Note: 100 mL of ICS solution can be prepared ahead of time; 1 mL aliquots should be kept frozen at -20 °C. For each experiment, thaw 1 mL of ICS solution and add fresh 0.5% biocytin (0.005 mg) and Lucifer Yellow (10 μg/mL). (Note that Lucifer Yellow is added to the patch-pipette to monitor the outflow.) Before each experiment, filter the ICS solution using a 0.22 μm filter unit (see laboratory supplies).*

**Table:** 

Reagent	Final concentration	Quantity or Volume
KCl	30 mM	0.22368 g
EGTA	5 mM	0.19018 g
HEPES	10 mM	0.23831 g
MgCl_2_	1 mM	0.00952 g
Na_2_ATP	3 mM	0.16534 g
CaCl_2_	0.5 mM	0.00555 g
Potassium gluconate	100 mM	2.34246 g
dH_2_O	n/a	Q.S. 100 mL
Total	n/a	100 mL

**5. Fixation solution with paraformaldehyde**

*Note: After adding paraformaldehyde to the PBS, slowly adjust the pH to 7.4 by adding NaOH dropwise. Aliquot and store at -20 °C.*

**Table:** 

Reagent	Final concentration	Quantity or Volume
Paraformaldehyde	40 g/L	40 g
Phosphate buffered saline	n/a	Q.S. 1 L
Total	n/a	1 L

**6. Blocking solution for immunohistochemistry**

*Note: Store at -20 °C*

**Table:** 

Reagent	Final concentration	Quantity or Volume
Triton X-100	2 mL/100 mL	2 mL
Bovine serum albumin	2 mg/100 mL	2 mg
Normal donkey serum	5 mL/100 mL	5 mL
Tris-buffered saline	n/a	Q.S. 100 mL
Total	n/a	100 mL

**7. Antibody incubation solution for immunohistochemistry**

*Note: Store at -20 °C.*

**Table:** 

Reagent	Final concentration	Quantity or Volume
Triton X-100	1 mL/100 mL	1 mL
Bovine serum albumin	1 mg/100 mL	1 mg
Normal donkey serum	2.5 mg/100 mL	2.5 mg
Tris-buffered saline	n/a	Q.S. 100 mL
Total	n/a	100 mL

**8. Washing solution for immunohistochemistry**

*Note: Store at -20 °C.*

**Table:** 

Reagent	Final concentration	Quantity or Volume
Bovine serum albumin	1 mg/100 mL	1 mg
Tween 20	0.1 mL/100 mL	0.1 mL
Tris-buffered saline	n/a	Q.S. 100 mL
Total	n/a	100 mL

**Laboratory supplies**

1. 2.5 mL SafeSeal reaction tubes (Sarstedt, catalog number: 72.695.400)

2. 1.5 mL Eppendorf tubes (Sarstedt, catalog number: 72.706.400)

3. Millex 0.22 μm filter unit (Merck Millipore, catalog number: SLGV004SL)

4. 24-well plates (Sarstedt, catalog number: 83.3922)

5. 400 mL glass beaker (fitted with six transparent PET cell culture inserts with nylon stockings glued to the bottom to serve as “chambers” for brain slices) (Carl Roth, catalog number: X692.1)

6. Transparent PET cell culture inserts (Falcon, catalog number: 353090)

7. 100 mL glass beaker (fitted with one transparent PET cell culture insert with nylon stockings glued to the bottom to serve as “chambers” for brain slices) (Carl Roth, catalog number: X689.1)

8. X10 double-sided razor blades (for vibratome) (Fisher Scientific, catalog number: 12043029)

9. Decapitation scissors (Fine Science Tools, catalog number: 14200-21)

10. Standard dissection kit (Deutsche Biomedical, catalog number: DBK1016)

11. Pasteur pipettes (Carl Roth, catalog number: EA70.1)

12. Filter paper (Carl Roth, catalog number: L875.1)

13. Tygon standard hoses S3 ID 1.6 mm × OD 3.2 mm (Carl Roth, catalog number: E-3606)

14. Glass Petri dish 10 cm Ø (Medplus catalog number: 54693)

15. Reusable blades (Carl Roth, catalog number: CK07.1)

16. Superglue (“Sekundenkleber” from UHU)

17. 20 μL micro loader pipette tips (Eppendorf, catalog number: 5242 956.003)

18. 1 mL syringe Braun Omnifix F Luer Solo (B Braun, catalog number: 9161406V)

19. Platinum harp slice anchor, custom-made in-house, but equivalents can be purchased from e.g., Warner instruments or NPI electronic

20. Borosilicate glass, 1.5 mm outside diameter, 0.315 mm wall thickness (Hilgenberg, catalog number: 1409249)

21. 15 mL centrifuge tubes (Biozym, catalog number: LC0052)

22. Glass Pasteur pipettes, length 150 mm (Fisher Scientific, catalog number: 1154-6963)

23. Disposable needles Terumo 0.9 × 38 mm (CLS Medizintechnik, catalog number: AN*2038R1)

24. Brush (TH.Geyer, catalog number: D770000)

25. Superfrost plus 25 × 75 × 1.0 mm glass slides (Epredia, catalog number: AB000008032ED1MNZ50)

26. Absorbing tissue, KIMTECH, Science Precision tissue, 7552, 286 pieces (Carl Roth, catalog number: AA64.1)

27. Cryotags (Carl Roth, catalog number: X547.1)

28. Coverslips, 24 × 40 mm (Carl Roth, catalog number: 1870)

29. Slide folder (Carl Roth, catalog number: AXK6.1)

30. Aqua Polymount mounting medium (Polysciences, catalog number: 18606-5)

## Equipment

1. Vibratome (Microm International GmbH, model: HM 650V)

2. Osmometer (Gonotec GmbH, model: Osmomat 3000 basic)

3. Incubator (Melag Medizintechnik, model: Type A)

4. Pipette puller (Sutter Instrument, model: P-97)

5. Anti-vibration air table (Microplan Schwingungstechnik)

6. Microscope (Slicescope II, Scientifica Ltd., or Axioskop 2 FS Plus, Carl Zeiss Microscopy GmbH)

7. CCD camera for microscope (Imago SensiCam, model: PCO AG)

8. Fluorescent light source VSG HBO 100/001.26E (JENA GmbH, catalog number: 2029)

9. Peristaltic pump for ACSF superfusion (Minipuls 3, AbiMed Gilson, Inc. or Periflo Flocon 1003, PetroGas Ausrüstungen Berlin GmbH)

10. 5× objective (Olympus, model: MPLFLN)

11. 60× water immersion objective (Olympus, catalog number: 1-U2B893)

12. Monochromator (Polychrome IV, Till Photonics GmbH, catalog number: 5855)

13. Filter set for SR101 (excitation and emission wavelengths of 555 and 585 ± 10 nm) (Leica)

14. Filter set multi-band XF53 (Omega Optical)

15. EPC 10 patch-clamp amplifier (HEKA Elektronik, catalog number: 895277)

16. Micromanipulator (Patch Star micromanipulator, Scientifica)

17. Patch-clamp control panels (Patch Star, Scientifica)

18. Stuart gyro-rocker SSL3 (Sigma-Aldrich, catalog number: Z654515)

19. Centrifuge (Eppendorf, model: 5417R)

20. Leica DM TCS SPE confocal microscope (Leica Microsystems GmbH, model: HC APO 20×/0.75)

## Software and datasets

1. TIDA 5.25 (HEKA Elektronik); license required

2. Igor Pro 7 (WaveMetrics); license required

3. LCS Lite or LAS AF Lite (Leica); license required

4. Fiji/ImageJ (NIH); free to use

5. Prism 6.07 and 10.1.2 (GraphPad); license required

6. CamWare for HSFC-pro 3.07 (PCO AG); license required

## Procedure

**A. Brain slice preparation**

Before slicing, prepare the solutions, vibratome, and surgical equipment. Slicing is a time-sensitive process as tissue quality degrades over time. Solutions must be made in advance with sufficient time to allow for carbonation and cooling/heating to the correct temperature.

1. Prepare required solutions

a. Prepare 500 mL of slicing solution (see Recipes), cool to 4 °C, and carbonate (95% O_2_, 5% CO_2_). Place the slicing solution in the slicing chamber right before beginning.

b. Prepare 1 L of ACSF (see Recipes) and carbonate (95% O_2_, 5% CO_2_). Place 250 mL of ACSF in a 300 mL beaker with nylon chambers (see Laboratory supplies) where brain slices will be stored.

c. Prepare 1 L of ACSF with SR101 (see Recipes) or thaw it from frozen stock solution if previously prepared. Carbonate (95% O_2_, 5% CO_2_). Place 75 mL of ACSF + SR101 in a small 100 mL glass beaker with a nylon chamber (see Laboratory supplies) where brain slices will be placed for incubation. Place the beaker in the incubator to let the solution warm up to 35 °C.

*Note: All solutions from steps A1a–c should be carbonated at least 15 min before use.*

2. Prepare vibratome and surgical equipment

a. Switch on the vibratome and mount a fresh X10 double-sided razor blade into the blade holder. Caution: handle sharps with care. Set the following settings on the vibratome: thickness = 250 µm; frequency = 90; amplitude = 1.0.

b. Prepare surgical equipment: scissors for decapitation, mini-scissors, tweezers, spatula, blade, and Pasteur cut pipette.

c. Prepare a glass Ø 10 cm Petri dish by filling it with ice. Place a cut-out piece of filter paper on top of the ice.

3. Prepare forebrain slices on the vibratome

*Note: This step is time-sensitive and should be carried out in a timely manner. Some protocols call for transcardiac perfusion with ACSF prior to decapitation and brain dissection. While not strictly necessary, this additional step may be appropriate if adapting the protocol for other brain regions or organs. Make sure to comply with local laws and regulations regarding the anesthesia and euthanasia of laboratory animals.*

a. Sacrifice mouse by cervical dislocation and sever head with large decapitation scissors.

b. Remove the brain from the skull, prepare it for slicing, and mount it on the vibratome. Cut open the skin with small scissors to expose the skull, starting from the base of the head (caudal/posterior) toward the nose (rostral/anterior).

c. Make two parallel cuts in the skull from the caudal side of the skull toward the nose on either side of the head using the small scissors. Make one cut arching to the right and one arching to the left so that the cuts meet at the frontal side of the skull between the eyes.

d. Carefully peel away the skull using forceps, gripping it by the caudal side.

e. Using a small spatula, remove the brain from the skull and place it in ice-cold slicing solution for a few seconds.

f. Remove the brain from the solution and place it on the filter paper of the chilled glass Petri dish. Slice off the brainstem using a reusable blade. Make an antero-posterior nick on one side of the cortex. This nick serves as a landmark to later identify how the slice was placed in the recording chamber during dye-filling. For staining and imaging, ensure that the slice always faces the same direction, with the filled network facing up.

g. Mount the brain onto the vibratome specimen holder using superglue with the frontal side pointing up.

h. Place the specimen holder with the brain into the slicing chamber of the vibratome.

i. Fill the vibratome slicing chamber with ice-cold slicing solution and continue to carbonate.

Caution: Follow local laws and regulations regarding biosafety when handling and disposing of animal tissue.

j. Using the settings specified in step A2a, cut 250 μm coronal brain slices of the mounted brain until the desired plane of the brain is reached. If interested in caudal structures, one might first make larger cuts through the rostral portion of the brain and discard these thicker slices. When the region of interest is reached, carefully remove each brain slice using a cut Pasteur pipette and place them into the holding chamber containing carbonated ACSF at room temperature (RT). Slices can remain stored here for up to 5 h.

**B. Pre-labeling of astrocytes with SR101**

1. Place a single slice into the incubator containing carbonated ACSF + SR101 at 35 °C for 20 min. Note that each slice is incubated individually before being immediately used for patch-clamp and dye-loading. Do not incubate the next slice before dye-loading of the previous slice has been successfully completed.

**C. Patch-clamp and biocytin dye-loading of SR101-labeled astrocytes**

After pre-labeling with SR101, individual astrocytes can be dye-loaded with biocytin through the patch-clamp. Patch-clamp recordings serve as an additional tool to verify astrocyte identity. Setup preparation can be done during SR101 labeling of slices.

1. Prepare setup and solutions

a. Turn on all equipment and run ACSF at RT through the patch-clamp chamber using the peristaltic pump fitted with Tygon tubing.

b. Thaw intracellular solution (ICS) (see Recipes) if frozen or take from the fridge if already thawed. Filter once with a Millex 0.22 μm filter unit.

2. Take the slice from the incubation chamber using a cut glass Pasteur pipette and place it into the recording chamber of the patch-clamp setup. Anchor the slice to the recording chamber using a platinum harp grid with nylon threads.

3. Using the 5× objective, locate the region of interest by moving the station with the recording chamber with the Scientifica control panel.

4. Once positioned above the region of interest, switch to the 60× water immersion objective to locate individual cells. Astrocytes can be identified by their SR-101-fluorescence at excitation and emission wavelengths of 555 and 585  nm ± 10 nm by switching on the monochromator for the fluorescent light source. Identify a fluorescent cell to be targeted for dye-filling before moving the objective up in the z-direction using the head stage control panel. Do not move in the x-plane and y-plane from this point.

*Note: Try to select cells that are at least a few microns deep in the slice, as cells close to the surface are likely dead or damaged from the vibratome slicing process.*

5. Prepare pressurized pipettes with ICS:

a. Pull a pipette from borosilicate glass on the pipette puller. Pipettes should have a resistance of 4–8 MΏ for astrocytes. The manufacturer may provide a list of settings for different applications, e.g., Sutter Instruments’ “Pipette Cookbook” (available online here ).

b. Fill the glass pipette with 5–10 μL of ICS + biocytin (see Recipes) and mount it onto the electrode on the micromanipulator pipette holder stage.

c. Using a clean 1 mL syringe, add 0.1 mL of positive pressure onto the pipette to ensure an outflow of ICS.

d. Once in the bath and with the tip of the pipette in the field of view, you can visualize the outflow of ICS by switching to the filter for Lucifer Yellow, excitation at 495 nm, and emission at 510  ±  10 nm.

6. Approach the selected astrocyte

a. Lower the pipette into the bath slowly, without yet moving it onto the slice.

b. Using the HEKA amplifier and TIDA software, a sweeping protocol should be applied in which a single -10 mV voltage step is applied, i.e., a test pulse. Once the pipette is submerged, this test pulse should become visible and stable. Check the pipette resistance under “R-Mem” on the TIDA control panel. Locate the pipette under the 60× objective by moving it into the center of the frame without lowering it onto the slice.

c. Move the pipette slowly toward the slice with the micromanipulator control panel, keeping it in focus until it is directly above the focal plane of the slice.

d. Switch on the fluorescent light source applying excitation at 495 nm and visualization at an emission wavelength of 510 ± 10 nm to confirm continued outflow of ICS + Lucifer Yellow from the pipette.

e. Compensate for the offset (V0Auto on the TIDA control panel).

**Figure 1.**
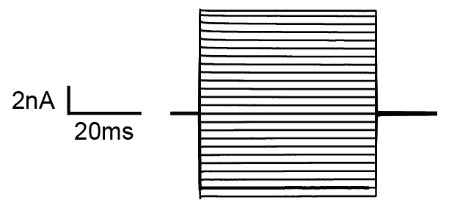
Typical current profile of an astrocyte in the hippocampus clamped at -70 mV in response to 10 de- and hyperpolarizing voltage steps. S R101 can also be taken up by oligodendrocytes and even neurons under certain conditions and—although it labels a large population of astrocytes—may not capture all astrocytes within a tissue [24].

**Figure 2.**
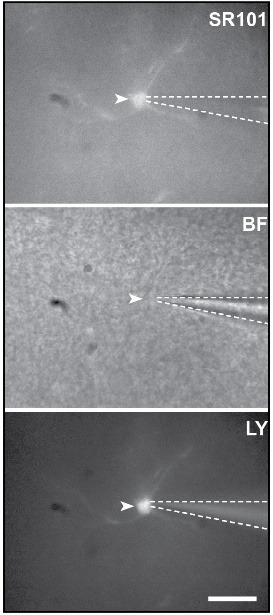
Fluorescence images of a patch-clamped astrocyte (white arrow) stained by Sulforhodamine 101 and Lucifer Yellow (LY) and a brightfield (BF, middle) view of the slice . To ensure the success of the dialysis, Lucifer Yellow was added to the pipette solution. Dotted line outline patch-pipette. Scale bar, 20 μm.

7. Patch and passively dye-fill the selected astrocyte

a. Position the pipette until it lightly touches the cell surface. This should be visible by a dimple on the cell membrane and an increase in pipette resistance by 0.2–0.4 MΏ. Cells may be moved by the positive pressure on the pipette (see Troubleshooting).

b. Remove the positive pressure and then slowly apply 0.05–0.2 mL of negative pressure. Pipette resistance should increase to several hundred MΏ within seconds. Monitor the pipette resistance until gigaseal (>1 gΏ) is reached.

c. Apply gentle and brief suction pulse with a syringe or by mouth to break into a whole-cell configuration. This should result in an abrupt increase in the amplitude of capacitative transients in response to -10 mV voltage steps.

d. Switch to an appropriate voltage step protocol to verify astroglial identity. We recommend recording membrane currents with a series of de- and hyperpolarizing voltage steps (10 mV each, filtered at 2.9 kHz) from a holding potential of -70 mV ranging from -160 to 50 mV for 50 ms. Analysis of the protocol and general features of the astrocytes are outlined below in data analysis. During recording, one can already view input resistance in TIDA, which for astrocytes is relatively low (< 50 MΏ). The current profiles displayed upon voltage stimulation are generally considered to be *passive*, i.e., lacking voltage-gated currents. For an example, see Figure 1.

e. Monitor leak-current and access-resistance over the course of 20 min, during which the cell is passively dye-filled. An abrupt change can indicate a loss of access to the cell.

f. At the end of 20 min, repeat the voltage clamp protocol described in step C7d. This can be used to determine stable cell access and cell health in downstream data analysis.

g. Confirm successful dye-loading of the cell by switching on the fluorescent light source and image Lucifer Yellow at excitation at 495 nm and visualization at an emission wavelength of 510  ±  10 nm. A clear fluorescent signal should be produced by the cell (Figure 2).

8. Slice removal and fixation

a. Carefully remove the pipette from the cell in the x and z planes by moving the micromanipulator with the control panel. To minimize the chance of pulling the patched cell out of the tissue alongside the pipette, you may try to first disrupt the seal by applying a large voltage step [33].

b. Carefully note on which side of the slice the nick is present. The slice should later be mounted onto a glass slide and imaged with the same side facing upward as in the recording chamber since the filled network will be closer to the exposed surface of the slice.

c. Carefully remove the slice from the recording chamber using a small brush and place it into a 2.5 mL Eppendorf tube filled with ACSF. Under a fume hood, transfer the slice to a 2.5 mL Eppendorf filled with 1.5 mL of paraformaldehyde fixation solution (see Recipes).

d. Leave it in paraformaldehyde fixation solution for 24 h at 4 °C.

Caution: Paraformaldehyde is toxic.

**D. Immunohistochemistry of dye-filled slices: Primary antibody incubation**

Fixated slices can be stained for biocytin-labeled networks and immunolabeled with antibodies targeting proteins of interest. To identify astrocytes, we recommend anti-GFAP antibodies, but alternatives may be used (see General notes).

1. Prepare the required solutions (blocking and antibody incubation solutions) if not previously prepared. These solutions can be aliquoted into 15 mL centrifuge tubes and frozen for future experiments. If already prepared, thaw enough volume of each. Per well/slice you need:

a. 450 μL of blocking solution

b. 300 μL of antibody incubation solution

2. Wash paraformaldehyde fixed dye-filled slices

a. Take all dye-filled slices out of the fridge. Under a fume hood, transfer slices to a 24-well plate filled with TBS using a small brush, placing a single slice in each well. When placing the slices into the TBS in the wells, keep the cut on the same side as it was during dye-filling. This ensures the antibodies can more readily access the top of the slice. Caution: Paraformaldehyde is toxic; take care to follow local laws and regulations regarding biosafety when disposing of toxic waste.

b. Take the 24-well plate out of the fume hood and place it on the gyro-rocker at RT at a speed of 10 rpm.

c. After 10 min, remove the TBS with a glass Pasteur pipette and exchange it for fresh TBS. Put it back on the shaker. Repeat once.

3. Blocking of nonspecific binding of primary antibodies

a. Remove the TBS and add 450 μL of blocking solution (see Recipes) to each well. Place it on the gyro-rocker at a speed of 10 rpm at RT for 2 h to permeabilize the tissue and block nonspecific binding of the primary antibodies.

4. Prepare primary antibodies in antibody incubation solution (see Recipes):

a. Calculate the total volume of incubation solution + antibodies needed. Per well/slice, you need 300 μL of antibody incubation solution + antibodies. Number of wells × 300 μL = total volume (μL).

b. Calculate the volume of primary antibodies that need to be added to the antibody incubation solution. Primary antibodies can vary based on the researcher’s interest. For the anti-GFAP antibody used in the present protocol:

Guinea pig anti-GFAP antibodies (1:500). Total volume in μL/500 = amount of antibody in μL.

*Note: You may stain for whichever other protein is of interest and use streptavidin coupled with any other fluorophore as appropriate (see General notes). Refer to the manufacturer or literature to determine appropriate antibody concentrations.*

c. Calculate the amount of antibody incubation solution needed: total volume – volume of both antibodies = volume of antibody incubation solution.

d. Transfer the calculated volume of antibody incubation solution to a reaction tube. Add the calculated volume of antibodies to the solution.

5. Primary antibody incubation

a. After 2 h of blocking, remove the blocking solution from each well using a glass pipette, taking care not to damage the slice.

b. Add 300 μL of antibody incubation solution + antibodies to each well.

c. If bubbles appear in the well after adding the solution, carefully pop them using a small disposable needle.

d. Place the well-plate on a gyro-rocker at a speed of 10 rpm at 4 °C and leave for 48 h.

**E. Immunohistochemistry of dye-filled slices: secondary antibody incubation/ biocytin labeling**

1. Wash the primary antibodies from the dye-filled slices

a. Prepare or thaw washing solution. Per well, you need 3 × 500 μL of washing solution (see Recipes)

b. Remove the primary antibody incubation solution from the wells using a glass Pasteur pipette, taking care not to damage the slice.

c. Add 500 μL of washing solution to each well and leave on the gyro-rocker at RT for 10 min at a speed of 10 rpm. Remove the washing solution after 10 min and repeat this step another 2 times for a total of 30 min of washing.

2. Prepare secondary antibodies in antibody incubation solution

a. Thaw antibody incubation solution.

b. Calculate the total volume of antibody incubation solution + antibodies needed: Number of wells × 300 μL = total volume.

c. Calculate the volume of secondary antibodies and DAPI that need to be added to the antibody incubation solution. In the present protocol:

i. Alexa Fluor 647-conjugated donkey anti-guinea pig IgG (1:200). Total volume in μL/200 = amount of antibody in μL.

ii. Cy3-conjugated streptavidin (1:200) to label biocytin. Total volume in μL/200 = amount of antibody in μL.

iii. DAPI (1:200). Total volume in μL/200 = amount of DAPI in μL.

*Note: You may instead use a mounting medium containing DAPI or add DAPI directly to the mounting medium, e.g., Aqua polymount (1.5 μL of DAPI in 5 mL).*

Caution: DAPI is toxic.

d. Calculate the amount of antibody incubation solution needed: total volume – volume of secondary antibodies + DAPI = volume of antibody incubation solution.

e. Centrifuge the secondary antibodies prior to use at 17,709× *g* for 15 min at 4 °C, making sure to protect from light.

f. Add the calculated volume of antibody incubation solution, the secondary antibodies, and DAPI to a 15 mL reaction tube. Protect from light by covering the tube in aluminum foil.

3. Secondary antibody incubation:

a. After the final washing step, remove the washing solution from each well.

b. Add 300 μL of antibody incubation solution + secondary antibodies + DAPI.

c. Place the well-plate on the gyro-rocker at RT at 10 rpm for 2 h.

4. Wash the secondary antibodies from the dye-filled slices

a. Remove the solution from each well.

b. Wash three times for 10 min each with washing solution on the shaker at RT.

c. Remove washing solution and add TBS.

5. Mounting slices on glass slides

a. Prepare a Ø10 cm glass Petri dish by filling the bottom of the dish with PBS. Place a piece of dark cloth or plastic underneath to visualize slices better.

b. Prepare glass slides by labeling them appropriately.

c. Place a glass slide with one half submerged in PBS in the Petri dish. Using a small paint brush, remove the first slice from the well plate and place it in the PBS. Using the brush, carefully move the PBS to gently place the slice onto the glass slide. Take care to manipulate the surrounding PBS rather than the slice itself as much as possible. Make sure the slice is mounted onto the glass slide with the cut on the correct side so that the dye-filled cells point upward.

d. Once mounted, remove the glass slide from the PBS and, using Kimtech absorbent tissue, carefully remove as much PBS as possible without touching the slice.

e. Cut two small sections of cryotags and place them on both sides of the brain leaving a space of 0.5 cm between the brain and the sticker. Repeat steps E5c–e for each brain slice.

f. Once the area around the brain slice is sufficiently dried, place one drop of Aqua Polymount. If any bubble appears, pop it with a small disposable needle. Carefully place a coverslip on top of the slice. Repeat for each brain slice.

g. Place all slides into a folder laying flat and place in the fridge at 4 °C for at least 24 h before scanning on the microscope.

**F. Data acquisition and analysis**

To determine the inclusion of dye-filled networks based on electrophysiological criteria, analyze the voltage protocols obtained at the start and end of 20 min of dye-filling using IGOR Pro 7 software, custom code [27], or similar software. Determine the basic membrane properties (i.e., input resistance, capacitance, reversal potential) from the voltage protocol run at the start of dye-filling. Typically, astrocytes have a low input resistance (<50 MΏ), a reversal potential of −70 to −80 mV, and linear IV curves. Perform an outlier analysis (ROUT, Q = 1%) on the basic membrane properties using e.g., GraphPad Prism and exclude outliers from the analysis. Determine series resistance from the voltage protocol at the start and end of the 20 min of dialysis and exclude all cells showing >125% increase in series resistance, as this indicates that access to the cell might have been compromised during the 20 min of dialysis and dye-filling has thereby become less reliable.

**Figure 3.**
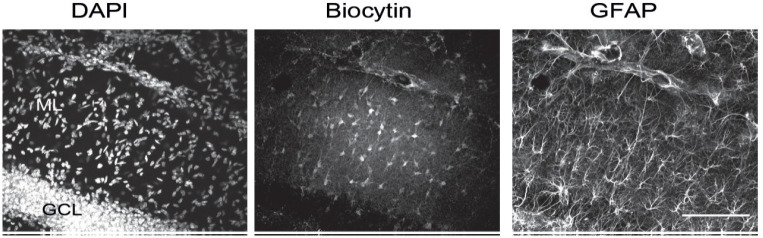
Z-stack images of DAPI, biocytin, and GFAP staining of a hippocampal slice in which an individual astrocyte was labeled by biocytin. The middle panel shows the labeling of many dye-coupled cells. GCL, granular cell layer; ML, molecular layer. Scale bar, 100 μm.

Because of the thickness of the slices and the possibly large extent of cell networks in the z-axis, imaging of dye-filled networks should be carried out on a confocal microscope such as the Leica DM TCS SPE. Briefly, using 5× magnification at excitation and emission wavelengths of 554 and 566 nm to visualize the Cy3-streptavidin signal, locate the network and place the objective at the center. Switch to the 20× objective and record a z-stack of the network from the deepest plane to the shallowest plane in which you are able to detect dye-filled, Cy3-positive cells. The step size between z-planes should be 1 μm, and the number of imaged planes in each stack will vary from slice to slice depending on the extent of dye diffusion. Adjust other settings (laser power, gain, etc.) as appropriate, and image all other fluorescent channels you wish to visualize, e.g., DAPI and GFAP immunostaining. To determine the size of dye-filled networks, images, e.g., Figure 3, can be analyzed with Fiji/ImageJ software using the cell counter plugin and z-axis projection functions.

## Validation of protocol

This protocol or parts of it has been used and validated in the following research articles:

- Pelz et al. [42]. The IgCAM BT-IgSF (IgSF11) is essential for connexin43-mediated astrocyte-astrocyte coupling in mice. eNeuro (see Figure 5 and extended Figure 5–1)
- Kompier et al. [17]. Membrane properties and coupling of macroglia in the optic nerve. Curr Res Neurobiol (see Figure 5)
- Philippot et al. [39]. Astrocytes and oligodendrocytes in the thalamus jointly maintain synaptic activity by supplying metabolites. Cell Reports (see Figure 3)
- Meyer et al. [16]. Oligodendrocytes in the Mouse Corpus Callosum Maintain Axonal Function by Delivery of Glucose. Cell Reports (see Figures 3 and 4, and supplementary Figures 3 and 5)
- Claus et al. [13]. Barreloid Borders and Neuronal Activity Shape Panglial Gap Junction-Coupled Networks in the Mouse Thalamus. Cerebral Cortex (see Figures 2, 3, 6, and 7)
- Griemsmann et al. [12]. Characterization of Panglial Gap Junction Networks in the Thalamus, Neocortex, and Hippocampus Reveals a Unique Population of Glial Cells. Cerebral Cortex (see Figures 1, 2, 4, 5, 6, and 7, and supplementary Figures 1 and 2)
- Richter et al. [14]. Glioma-associated microglia and macrophages/monocytes display distinct electrophysiological properties and do not communicate via gap junctions. Neuroscience Letters (see Figure 3)
- Maglione et al. [43]. Oligodendrocytes in mouse corpus callosum are coupled via gap junction channels formed by connexin47 and connexin32. Glia (see Figures 1 through 6)

## General notes and troubleshooting

**General notes**

1. This protocol builds on other protocols previously used for both glial cells and neurons. In fact, dialysis of biocytin or other tracers and dyes via electrode or patch-pipette will be intimately familiar to any electrophysiologist who has carried out morphological reconstruction of neurons (see, e.g., [28–31]). The use of electrodes or patch-pipettes to label gap-junction coupled glial cells can also be traced back to the 1980s and 1990s (see, e.g., [32–36]). We simply wish to provide a formalized step-by-step guide on how to implement it as a resource for the community.

2. The most technically challenging aspect of this protocol is patch-clamp electrophysiology, and aside from the confocal microscope, it is also the costliest in terms of equipment. Implementing this protocol should therefore be relatively trivial for any lab that already carries out patch-clamp electrophysiology experiments and possesses the requisite equipment and expertise. For labs in which this is not the case, it may be most sensible to establish a collaboration to carry out this protocol rather than attempting to implement it in-house unless there are additional reasons to be setting up a patch-clamp electrophysiology capacity.

3. The parameters described in the present protocol were optimized to label murine astrocyte networks in forebrain grey matter, specifically in the cortex, hippocampus, and thalamus. Its application in other brain regions or in other cell types will certainly require adjustments. SR101 can also be taken up by oligodendrocytes and even neurons under certain conditions and—although it labels a large population of astrocytes—may not capture all astrocytes [24]. For example, under specified conditions, SR101 incubation would lead to nonspecific staining in white matter or otherwise highly myelinated regions, rendering astrocyte identification impossible. Incubation parameters could conceivably be adjusted to mitigate this (i.e., lower temperature or shorter incubation times) but this would have to be validated. In this case, the use of transgenic mice expressing cell type–specific fluorescent reporters may be advisable.

4. Likewise, the general principle of this protocol would certainly be applicable to other species, tissues, and cells exhibiting gap-junction-mediated coupling, but the specific parameters have to be tested and validated on a case-by-case basis. In fact, some early examples of dye-loading to visualize gap junction–mediated coupling between cells were carried out in fish blastomere and turtle retina [37,38].

5. Selection of the tracer or dye to dialyze will similarly depend on the specific experiment you are carrying out, but four basic conditions have to be met: the tracer has to be water-soluble, non-toxic, of small molecular weight (<500 Da), and amenable to chemical fixation by aldehydes. Sulforhodamine 101 fulfills the first three but not the last; it is therefore washed away and no longer present in the fixed, coverslipped slide. Lucifer Yellow fulfills all conditions and has historically been used for this purpose, but biocytin has the advantage of amplification of the signal via fluorophore-conjugated streptavidin binding. Alexa Fluor dyes may be a viable alternative.

6. GFAP antibodies may be used if the researcher wants to study astrocytes incorporated in networks. Since oligodendrocytes may also be part of coupled glial networks [11,13,39], these cells may also be identified by labeling oligodendrocyte-specific proteins, such as Olig2 [12]. Not all astrocytes may be captured using GFAP immunolabeling. Particularly in the cortex, GFAP is known to primarily label endfeet rather than somata, and other astrocyte-specific proteins, like S100β and glutamine synthetase, may be targeted as an alternative [40,41] [Rabbit anti-Olig2 (Millipore, catalog number: AB9610), Mouse anti-glutamine synthetase (Millipore, catalog number: Mab302), Rabbit anti-S100β (Epitomics, catalog number: 2017-1)].

7. For quantification of the number of dye-coupled cells, we recommend using automated, unbiased tools such as Fiji/Image J’s cell counter plug-in or equivalent alternatives. If you cannot avoid counting cells “by eye,” it is crucial to ensure the person counting is blinded to the experimental conditions of samples and that a single experimenter does the counting for all samples in a given experiment or study, as there is considerable variation in the subjective threshold in fluorescence any one person may consider to warrant inclusion or exclusion.

8. It is important to consider that the visualized networks may in fact only represent a portion of the actual glial network in the tissue due to 1) the finite amount of biocytin in the patch-pipette, 2) the finite amount of time given for dialysis (in this case, 20 min), and 3) the decreasing concentration of biocytin as it diffuses further and further from the initially dialyzed cell. Assessing the size of networks therefore only makes sense if you seek to either determine their existence (i.e., is cell coupling present at all?) or are carrying out a comparison between two experimental conditions (i.e., between a mouse model of disease and healthy controls), while all other parameters remain the same.

9. SR101 incubation parameters must be carefully adapted to different conditions, as overstaining and nonspecific staining may occur, for example in highly myelinated regions. Overstaining has to be tested experimentally by analyzing double staining of SR101 with astrocyte-specific marker proteins such as GFAP. Moreover, while dye-filling using biocytin will reveal dye-coupling of cells, the full size of the coupled network may be beyond the rate of diffusion. Although the current protocol is specifically designed for astrocytes in mouse brain tissue, tracer dialysis through patch-clamp can also be applied in other cell types, tissues, and animal models that exhibit gap junction–mediated cell-to-cell coupling.

**Troubleshooting**

Problem 1: Overstaining by Sulforhodamine 101.

Possible causes: Temperature variation, slice thickness variation, prolonged incubation, regional variations.

Solution: If the protocol is precisely followed using the same region, slice thickness, incubation time, and temperature, no overstaining should occur. If using thinner slices with a higher incubation temperature and longer incubation times, then overstaining is likely. Additionally, this protocol is intended for forebrain regions and has been verified in the hippocampus, cortex, and thalamus. Regional differences may influence SR101 uptake. When overstaining occurs using a different region under these experimental parameters, try a shorter incubation time or lower the incubation temperature.

Problem 2: Frequent clogging of patch-pipette.

Possible causes: Air bubbles, unclean preparation of patch-pipettes, unclean ICS, loss of positive pressure, and contaminants in ACSF and/or recording chamber.

Solution: Unobstructed outflow of ICS can be confirmed by visualization of Lucifer Yellow out of the pipette. Sometimes, outflow is not visible, and instead, small particles or bubbles are visible inside the pipette. Formation of air bubbles may be prevented by shaking the patch-pipette downward before mounting it onto the micromanipulator. Small bubbles also tend to be pushed out of the patch-pipette through the positive pressure and will thus resolve themselves when waiting a few minutes. If the patch-pipette is frequently clogged with particles when entering the bath ACSF, the patch-pipette may have been contaminated during pulling or mounting in unclean conditions. Make sure that borosilicate glass for pulling pipettes is always stored in a clean and closed space. Before placing borosilicate glass into the pipette puller, wipe the glass down with a Kimtech tissue. Wear gloves during the preparation and mounting of pipettes. Alternatively, small flecks of dirt or dust may be introduced into the ICS upon repeated use. Filter the ICS through a Millex 0.22 μm filter before each experimental day and repeat again if clogging occurs. You may also need to rechlorinate the electrode and leave it in a patch-pipette filled with ICS overnight. Make sure that the ACSF, tubing, and recording chamber are free of dirt, debris, or contaminants and are cleaned regularly (we recommend running demineralized water through all tubing at the end of each experimental day and HCl every few weeks if all components of the rig are acid-resistant). Finally, if clogging occurs when approaching the cell but not when entering the pipette in the bath solution, you might be dealing with pressure loss (see Problem 3).

Problem 3: Pressure loss.

Possible cause: Leak in the system.

Solution: When outflow of ICS does not appear or becomes weaker over time, and no obstruction to the patch-pipette is visible, you might be dealing with pressure loss. This is additionally noticeable when approaching the slice and the pipette becomes clogged. Frequent places where leaks occur are in the tubing and in the O-rings of your patch-pipette holder. Demount the patch-pipette from the micromanipulator, including the tubing, and mount a glass pipette filled with ICS onto the electrode. Place the pipette holder and tubing into a bath of water and apply pressure. The appearance of bubbles will indicate the location of the leak. If in the tubing, replace the tubing. If coming from the pipette holder itself, replace the O-rings.

Problem 4: Noise.

Possible cause: Improper grounding, reference electrode not fully immersed.

Solution: If your traces look unstable or show regular oscillations, this is likely caused by 50 Hz noise from the main power line. Check whether all devices within the Faraday cage are properly grounded and whether your reference electrode is fully immersed in the bath solution.

Problem 5: Cells move during approach.

Possible cause: Cells are too superficial, slices are too old, intrinsic tissue properties.

Solution: It is expected for cells to slightly move during the approach of your patch-pipette due to the positive pressure. In this case, the approach takes time but is ultimately achievable. However, sometimes the cells move extensively and are thereby virtually impossible to patch. Cells that are on the most superficial layer of your slice may have become loosened during slicing. Aim for cells that are a bit deeper into the slice. Moreover, the quality of the slice may deteriorate over time in the recording chamber if many attempts have been made. After 1.5 h on one slice, if unsuccessful, discard it and try a new slice. How mobile cells are within the tissue will also depend on the specific location; for example, white matter is devoid of extracellular matrix, and the lipids in myelin tend to float upward since they are less dense than the water-based ACSF, making cells particularly likely to shift during approach.

Problem 6: Patched cell is removed when moving the patch-pipette out of the bath.

Possible cause: Abrupt movements.

Solution: When you consistently remove the patched cell along with your pipette at the end of the experiment, you might be removing your pipette too abruptly. Remove the pipette slowly by moving it in both z and x directions. Additionally, chances of pulling the cell are minimized by first disrupting the seal through the application of a large voltage step (see e.g., 33).
